# Predicted 3D Model of the Rabies Virus Glycoprotein Trimer

**DOI:** 10.1155/2016/1674580

**Published:** 2016-05-04

**Authors:** Bastida-González Fernando, Celaya-Trejo Yersin, Correa-Basurto José, Zárate-Segura Paola

**Affiliations:** ^1^Laboratorio de Medicina Traslacional, Escuela Superior de Medicina, Instituto Politécnico Nacional, Plan de San Luis y Díaz Mirón s/n, Santo Tomas, Miguel Hidalgo, 11340 Ciudad de México, DF, Mexico; ^2^Laboratorio de Biología Molecular, Laboratorio Estatal de Salud Pública del Estado de México, Paseo Tollocan s/n, La moderna de la cruz, 50180 Toluca, MEX, Mexico; ^3^Laboratorio de Modelado Molecular y Bioinformática, Escuela Superior de Medicina, Instituto Politécnico Nacional, Plan de San Luis y Díaz Mirón s/n, Santo Tomas, Miguel Hidalgo, 11340 Ciudad de México, DF, Mexico; ^4^Departamento de Bioprocesos, Unidad Profesional Interdisciplinaria de Biotecnología, Instituto Politécnico Nacional, Avenida Acueducto de Guadalupe s/n, Ticoman, Gustavo A. Madero, 07340 Ciudad de México, DF, Mexico

## Abstract

The RABVG ectodomain is a homotrimer, and trimers are often called spikes. They are responsible for the attachment of the virus through the interaction with nicotinic acetylcholine receptors, neural cell adhesion molecule (NCAM), and the p75 neurotrophin receptor (p75NTR). This makes them relevant in viral pathogenesis. The antigenic structure differs significantly between the trimers and monomers. Surfaces rich in hydrophobic amino acids are important for trimer stabilization in which the C-terminal of the ectodomain plays an important role; to understand these interactions between the G proteins, a mechanistic study of their functions was performed with a molecular model of G protein in its trimeric form. This verified its 3D conformation. The molecular modeling of G protein was performed by a I-TASSER server and was evaluated via a Rachamandran plot and ERRAT program obtained 84.64% and 89.9% of the residues in the favorable regions and overall quality factor, respectively. The molecular dynamics simulations were carried out on RABVG trimer at 310 K. From these theoretical studies, we retrieved the RMSD values from C*α* atoms to assess stability. Preliminary model of G protein of rabies virus stable at 12 ns with molecular dynamics was obtained.

## 1. Introduction

Rabies is a 100% fatal disease caused by the rabies virus (RABV) that affects the central nervous system [1]. Rabies virus belongs to the order Mononegavirales, classified in the Rhabdoviridae family, which includes at least three genera* Lyssavirus*,* Ephemerovirus*, and* Vesiculovirus*. The genus* Lyssavirus* includes rabies virus. The viral genome consists in a single and negative-stranded nonsegmented RNA, which encodes five proteins: nucleoprotein, matrix protein, phosphoprotein, glycoprotein, and the viral-dependent RNA polymerase [2, 3].

The glycoprotein (RABVG) rabies virus is comprised of four domains: signal peptide (SP), ectodomain (ED), transmembrane (TM), and a cytoplasmic domain (CD) [4, 5]. The RABVG is 65 kDa and has 524 amino acids. This is due to the presence of its signal peptide (SP) that is located on the N-terminal. It spans 19 residues. The SP is responsible for anchoring the protein to the ER-Golgi Apparatus (AP) membrane. This promotes subsequent transport of the nascent protein to the membrane before it is cleaved from the N-terminus in the AP [6, 7].

The RABVG in each peak is anchored in the plasma membrane and the lipid envelope by the transmembrane domain of 22 amino acids from 439 to 461 residues [8]. The C-terminal with the final 44 amino acids is the cytoplasmic domain. It extends into the cytoplasm of the infected cell where it interacts with M to complete the viral assembly [9].

On the other hand, the RABVG ectodomain is a homotrimer that contains a transmembrane domain. Each monomer has 439 residues, and the trimers are commonly called spikes. These spikes are responsible for the attachment of the virus through the interaction with nicotinic acetylcholine receptors, neural cell adhesion molecule (NCAM), and the p75 neurotrophin receptor (p75NTR). This makes them relevant for viral pathogenesis [10–15]. In addition, these receptors are responsible for the fusion of the viral envelope with the cell membrane as induced by a low pH [16]. This promotes the transsynaptic viral spread to the central nervous system. They can also act as targets for helper and cytotoxic T cells.

The RABVG C-terminal has 44 amino acid cytoplasmic domains that interact with the matrix protein to complete the viral assembly [9].

The RABVG protein induces an immune response due to its multiple antigenic domains. Hence, RABVG is the major contributor to RABV pathogenicity [17]. The antigenic structure differs significantly between the trimers and monomers. It has been reported that surfaces rich in hydrophobic amino acids are important for the trimer stabilization in which the C-terminal of the ectodomain plays an important role [18].

## 2. Material and Methods

### 2.1. Sequence Analysis, Modeling, and Stereochemical Analysis

This study was designed to predict the 3D model of RABVG protein under an iterative threading assembly refinement algorithm implemented in I-TASSER [19]. This was performed because the experimental 3D structure of RABVG protein was not available at Protein Data Bank (PDB) (http://www.rcsb.org). Various physical and chemical parameters of primary structure analysis were computed using the ProtParam online tool [20]. The secondary structure of the protein was computed using J-PRED servers [21]. The DiANNA tool [22] was used to check the system classification and disulfide connectivity. This knowledge can be helpful in understanding the secondary structure of the protein because the disulfide bond bridges are important in protein fold stabilization. The transmembrane topology of the RABVG was checked using TMHMM [23], MEMSAT3, and MEMSAT-SVM [24].

Finally, the 3D model of RABVG was generated using the I-TASSER online server [25]. This generated 3D models along with their confidence score (*C*-score). After generating the 3D model, structure and stereochemical analysis were performed using different evaluations and validation tools. The Psi/Phi Ramachandran plot was obtained using PROCHECK [26]. This assisted in the evaluation of backbone conformation. The Ramachandran plot was used to check the non-Gly residues in the disallowed regions. Structural quality of the model was assured using *Z*-scores, which indicate the overall model quality and confirm that the predicted structure is within the range of scores as found in the native proteins. The ProSA web tool [27] was used to determine the *Z*-scores. Furthermore, the generated model was submitted in the protein model database (PMDB) (https://bioinformatics.cineca.it/PMDB/) with PMDB identifier PM0079619.

With the monomer structure in hand, we attempted to make the trimer interact with protein-protein docking studies to predict the protein complex formed in a protein-protein interaction. These docking studies used the ClusPro server [28–31] that is the first fully automated web-based program for docking proteins. It was one of the top performers at CAPRI (Critical Assessment of Predicted Interactions) rounds 1–12—a community-wide experiment devoted to protein docking [28].

We used the PDBsum Generate server [32] to understand the interactions between—and assembly of—the five subunits of CRP. This server helps us analyze the interfaces between the subunits and summarizes the interactions across any selected interface. This server also provides information about the residues that actually interact across the interface.

### 2.2. Molecular Dynamics Simulations

These MD simulations employed NAMD 2.6 (Nanoscale Molecular Dynamics) [33] by applying the CHARMM27 force field for lipids and proteins [34]. This first neutralized the model RABVG trimer with 24 sodium ions along with the TIP3P model for the water box containing 58,566 waters molecules. Structural energy minimization was done using 10,000 steps. Multiple time-stepping algorithms were used with an integration time step of 2 fs. Various interactions were computed in 1, 2, and 4 time steps for covalent bonds, as well as short-range nonbonded interactions and long-range electrostatic forces, respectively. For every ten time steps, the nonbonded interactions had a pair list distance of 13.5 Å. The Van-der-Waals and electrostatic interactions were defined as interactions between short-range nonbonded interactions between particles within 12 Å. A smoothing function was employed for 10 Å Van-der-Waals interactions. Simulations were performed on the equilibrated system for 80 ns under constant pressure and a temperature of 1 atm and 310 K, respectively. The structure with the least energy and converged root mean square deviation was used for subsequent exercises. The final structure was analyzed with CARMA [35] and visualized with the VMD [36] program using 100 frames.

## 3. Results

### 3.1. Structural Description of the RABVG 3D Model

This study was initiated to perform structure-based sequence analysis studies on RABVG. The protein sequence was retrieved using accession AGN94258.1 from the NCBI protein. The primary structure analysis showed that the RABVG protein had a molecular weight of 58487.3 Daltons and a theoretical isoelectric point (pI) of 7.83. The instability index (II) is computed to be 38.05. This classifies the protein as stable. The negative grand average of hydropathicity (GRAVY) shows a value of −0.173 indicating that the protein was hydrophilic according to other reports [37].

**Figure 1:**
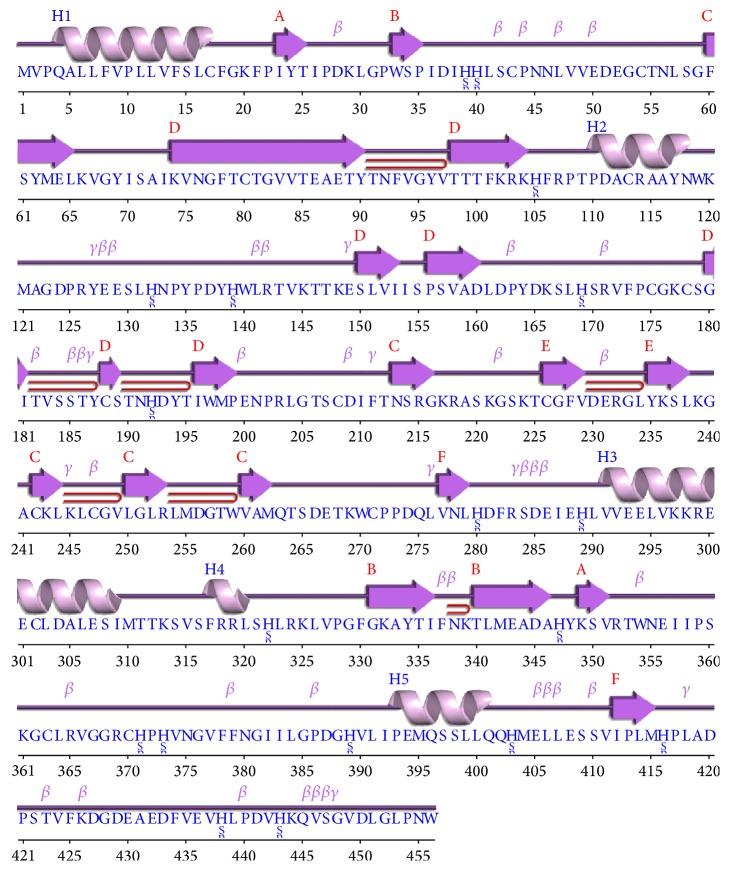
Predicted secondary structure of RABVG using the J-prep pserver.

Sequence and secondary structure analyses of RABVG revealed that it has 6 *β*-sheets, 7 beta hairpins, 3 beta bulges, 21 strands, 5 *α*-helices, 10 helix-helix interacs, 38 beta turns, and 10 *γ*-turns. Secondary structural features are shown in Figure 1. Disulfide bonds predicted by DiANNA are shown in Table 1.

**Figure 2:**
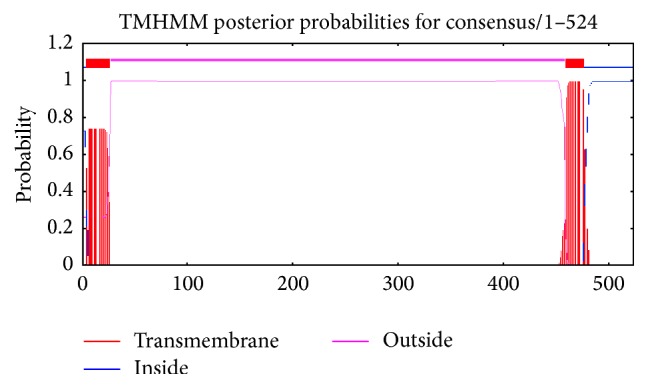
A transmembrane motif is revealed along with a 23-amino acid signal peptide at the extracellular N-terminus.

Disulfide connectivity was predicted to be within 1–8, 2–7, 3–12, 5–11, 6–10, 9–19, 13–17, 14–18, and 15-16. The TMHMM, MEMSAT3, and MEMSAT-SVM programs identify a transmembrane region between amino acids 460 and 480. The C-terminal is possibly located in the cytoplasm. The N-terminal—including 19 amino acids from the signaling peptide—is located in the extracellular region (Figure 2).

**Figure 3:**
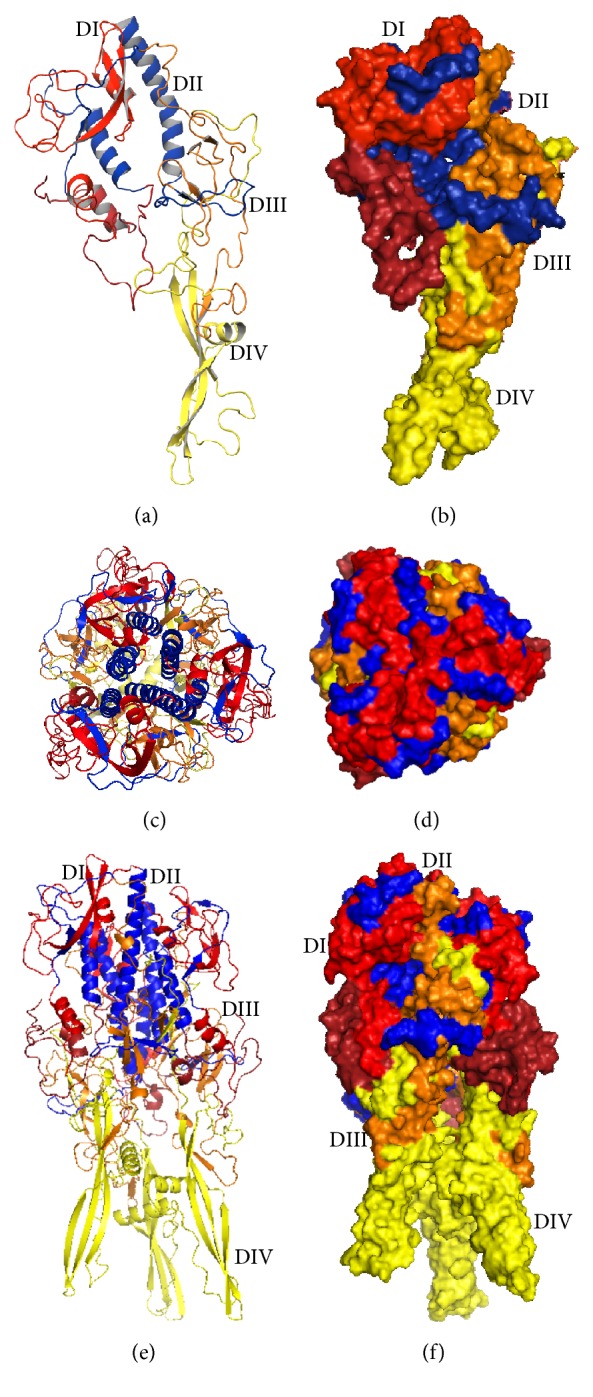
Domains of glycoprotein. The top lateral domain (DI) contains about 90 residues in two segments (1 to 17 and 312 to 383). Trimerization domain (DII) is made of three segments (18 to 35, 259 to 311, and 384 to 409), PH domain (DIII) is inserted within domain II. It is made of two segments (36 to 50 and 181 to 258) and has the fold of a pH domain. Fusion (DIV) (51 to 180) is inserted in a loop of the pH domain and is made of an extended sheet structure at the tip of which two loops are located that constitute the membrane-interacting motif of the G ectodomain. (a) Glycoprotein monomeric divided into 4 domains: DI (red) is lateral domain, DII (blue) is trimerization domain, DIII (orange) is domain of pH, and DIV (yellow) is fusion domain. (b) Surface representation of glycoprotein monomeric colored by domains. (c) Top view of the trimer, colored by domain, shows the formation of 6 alpha helices (blue) which contribute to the stability of the structure. (d) Top view of the trimer surface representation. (e) Glycoprotein trimer (divided into different domains) does not show a significant change in the organization of the domains. (f) Surface representation of the glycoprotein trimer, showing the cavity inside the molecule.

Knowing the 3D structure of RABVG is very important to understanding the proteins interactions, functions, and their important site localization. The model was not obtained by homology because the identities were low level (23%) whit 2J6J and 2CMZ crystals; for closely related protein sequences with identity higher than 40%, the alignment is almost always correct. Regions of low local sequence similarity become common when the overall sequence identity is below 40% [38, 39]. In this sense, the 3D structure of RABVG was predicted using the I-TASSER (http://zhanglab.ccmb.med.umich.edu/I-TASSER/) online server and the best predicted structure with the maximum confidence score (*C*-score −2.18) was selected (Figure 3 monomer) to achieve protein-protein docking studies using the Cluspro (http://cluspro.bu.edu/)server to get a trimer complex for visualizing the protein interactions (Figure 3).

The central trimerization domain has a significant contribution to the structural stability of the G protein trimer, due to the formation of 6 alpha helices. The different conformations (monomer, dimer, and trimer) of G protein are pH-dependent, several acidic residues (Glu286, Glu293, Glu294, Glu405, and Glu408) are brought close together in the postfusion bundle of six helices, suggesting that the acid residues play molecular switches role in their deprotonated forms, and this should destabilize the central six-helix bundle and thus allow the refolding of G back toward its prefusion conformation similar to VSV G protein [40].

These residues ensure a correct activation of G and ensure that the stability of the 6 chain helices has to be tightly regulated since both its destabilization and overstabilization are detrimental to the virus.

The glycoprotein of rhabdovirus is the target of neutralizing antibodies; its antigenicity and antigenic sites of G have been extensively studied in VSV and RABV [41]. The antigenic site II of RABVG is located between positions 34 and 42 and positions 198 and 200. These peptides are probably linked by a disulfide bridge and held together in the tertiary structure of G antigenic site III which extends from amino acids 330 to 338. This site is associated with the virulence [42].

They have described various regions of the protein G which have an important role in membrane fusion for the internalization of the virus; the region between amino acids 118 and 139 was generally considered to represent an internal fusion peptide for VSV G. However, other studies demonstrated that amino acids 395–418 have a significant influence on fusion, and additional studies identified region 145–164, termed p2-like peptide, as being a pivotal domain in facilitating glycoprotein G-mediated membrane fusion [43]. The stability and the preservation of these areas are important in the structure of G protein for viral internalization.

The structural organization of G is very similar to that of VSV G. This similarity extends from the N-terminal part to at least the end of helix G of domain II. It includes both the PH domain and the fusion domain (109 residues of the fusion domain), as well as the trimerization domain, and reveals a clear structural homology between the two proteins.

G has an altogether different structural organization from those of both class I and class II viral fusion proteins described so far. The polypeptide chain of G folds into four distinct domains (Figure 3): a lateral domain rich in *β* sheet at the top of the molecule (domain I), a central, mostly *α*-helical domain that is involved in the trimerization of the top of the molecule (domain II), a neck domain that has the characteristic fold of pleckstrin homology (PH) domains (domain III), and the elongated fusion domain that makes the trimeric stem of the molecule (domain IV). The C-terminal part of G corresponds to AA 411 to 422 in VSV and 410 to 455 in RAVBG [44].

The top lateral domain I contains about 90 residues in two segments (1 to 17 and 310 to 383 for VSV and 1 to 17 and 311 to 383 for RABV). Domain II is made of three segments (18 to 35, 259 to 309, and 384 to 405 for VSV and 18–35, 269 to 310, and 384 to 409 for RABV). Domain III is inserted within domain II. It is made of two segments (36 to 50 and 181 to 258 for VSV and RABV) and has the fold of a PH domain. Domain IV (51 to 180 for VSV and RABV) is inserted in a loop of the PH domain [44].

These residues ensure a correct activation of G and show that the stability of the 6 chain helices is tightly regulated because both its destabilization and overstabilization are detrimental to the virus.

**Figure 4:**
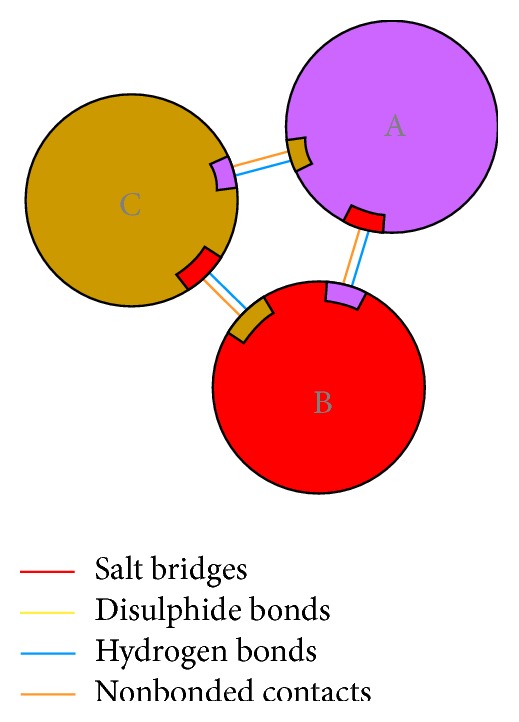
Schematic diagram showing the interactions between the subunits.

The number of residues involved in residue-residue interactions and a model of the interactions between each monomer denominated A-B, B-C, and C-A are shown in Figure 4. The model depicted in Figure 4 shows that the hydrogen bonds and other nonbonded interactions are responsible for the interactions among the monomers to build the trimer complex.

**Figure 5:**
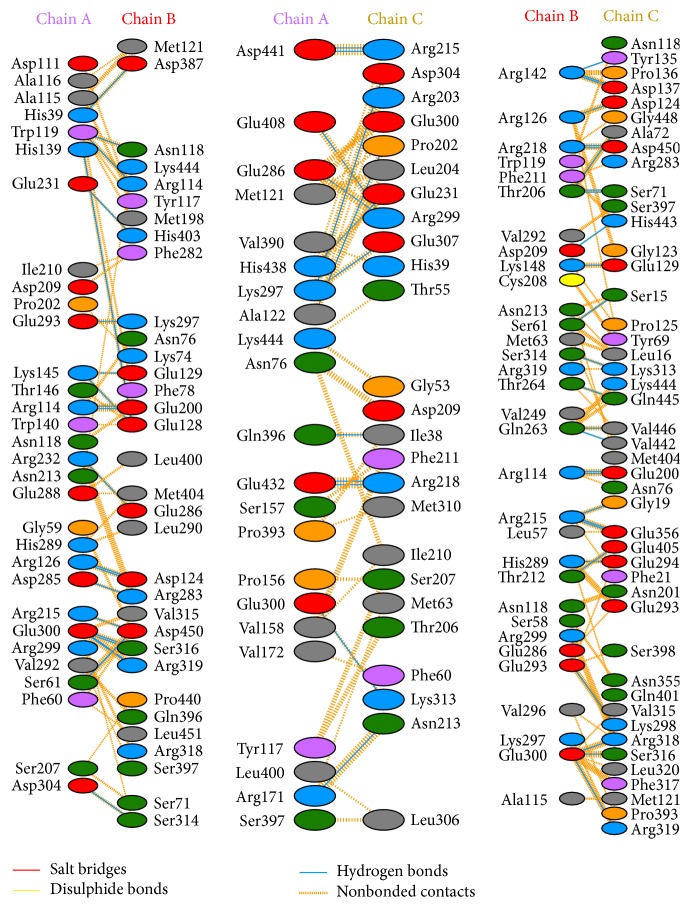
A model showing the number of interactions across the interfaces and the individual residue-residue interactions across the interfaces along with involved residues. Residue colors: positive (H, K, R) (blue); negative (D, E) (red); S, T, N, Q = neutral (green); A, V, L, I, M = aliphatic (gray); F, Y, W = aromatic (purple); P, G = Pro&Gly (orange); and C = cysteine (yellow). These include interactions between A and B interface, interaction between A and C interface, and interaction between B and C.

This trimer model shows the number of interactions across two interfaces as well as details of the individual residue-residue interactions across these interfaces (Figure 5).

**Figure 6:**
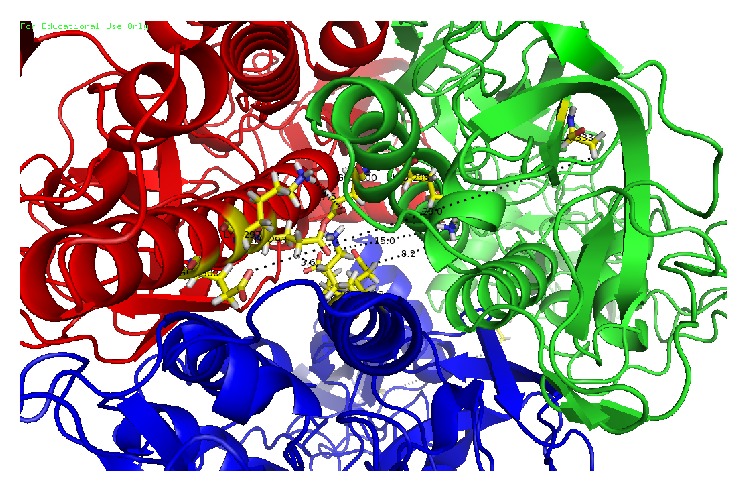
Glycoprotein trimer interactions (snapshot 80 ns): chain A (red), chain B (blue), and chain C (green); the interactions are shown between the chains, Glu 293 chain A with Lys 297 chain B, Glu 300 of chain A with Lys 313 of chain C, Lys 297 of chain A with Glu 300 of chain C, Lys 148 chain B with Glu 129 chain C, and Lys 298 chain C with Glu 286 and 293 B chain.

The interactions analysis in the 3D structure was obtained by a PDBsum server; for these hydrogen bonds and other nonbonded, disulfide bridges of CYS between two side chains or the formation of an amide bond (–CO–NH–) between side chains of Lys and a dicarboxylic aminoacid (Glu or Asp) were considered. The 3D structure presents electrostatic interactions between Glu 293 in chain A with Lys 297 in chain B, Glu 300 of chain A with Lys 313 of chain C, Lys 297 of chain A with Glu 300 of chain C, Lys 148 on chain B with Glu129 on chain C, and Lys298 on 25 chain C with Glu 286 and 293 on chain B. These stabilize the structure (Figure 6).

**Figure 7:**
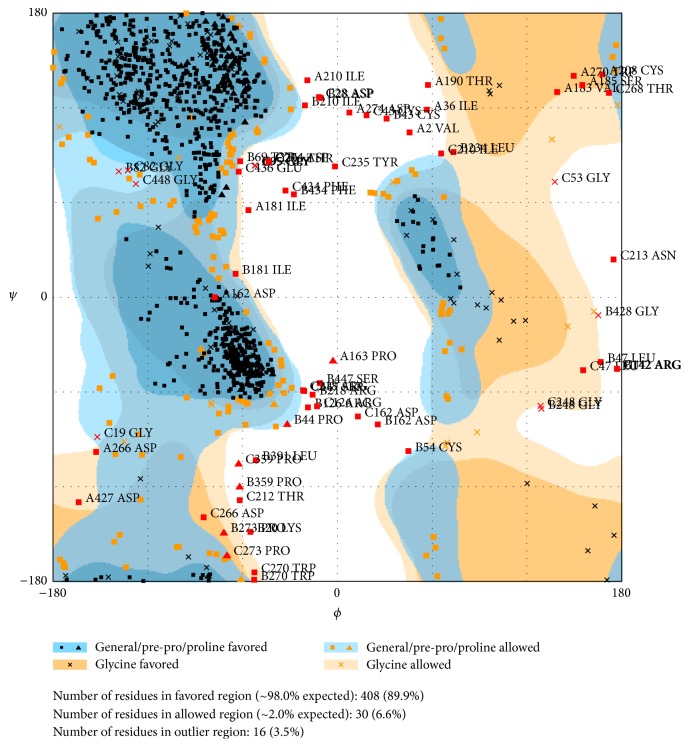
Ramachandran plot showing residues in the most favorable region and disallowed regions (RAMPAGE by Paul de Bakker and Simon Lovell available at http://mordred.bioc.cam.ac.uk/~rapper/rampage.php) [46].

Quality and reliability of the RABVG structure were checked using *Z*-score and Ramachandran plot. The stereochemical quality of the RABVG 3D structure was checked with a Ramachandran plot by analyzing the backbone dihedral angles residue by residue. The result showed that 89.9% of the residues were in the favorable region (Figure 7).

**Figure 8:**
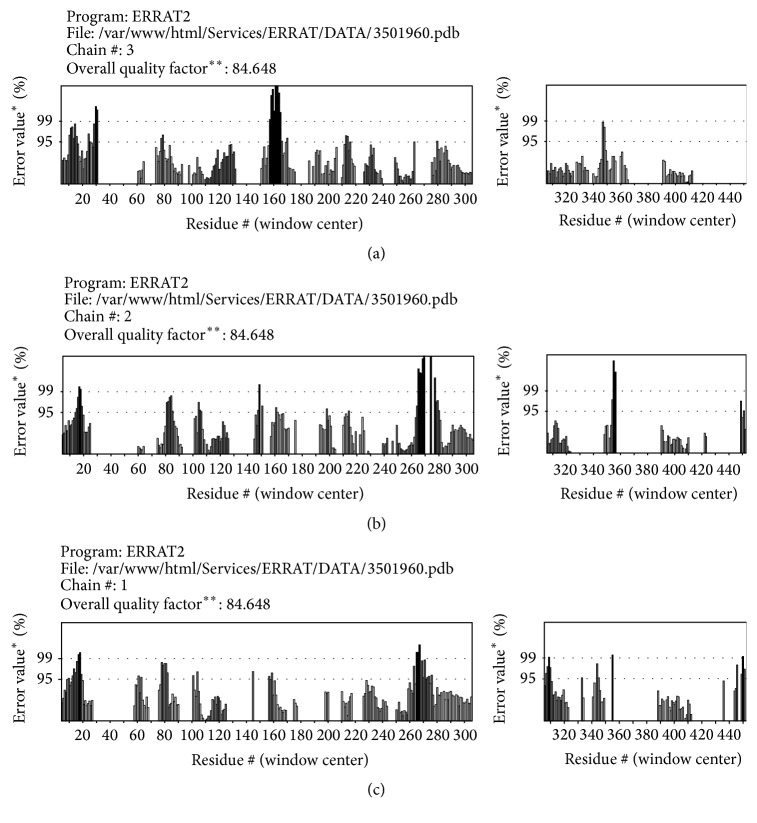
The ERRAT model had good overall quality. (a) Graphic of ERRAT program for A monomer. (b) Graphic of ERRAT program for B monomer. (c) Graphic of ERRAT program for the C monomer. ^*∗*^On the error axis, two lines are drawn to indicate the confidence with which it is possible to reject regions that exceed that error value. ^*∗∗*^Expressed as the percentage of the protein for which the calculated error value falls below the 95% rejection limit. Good high resolution structures generally produce values around 95% or higher. For lower resolutions (2.5 to 3 A) the average overall quality factor is around 91% [45].

The overall model quality can be checked with ProsA *Z*-score that is used to check whether the input structure is within the range of scores typically found for native proteins of similar size [27]. The *Z*-score of the protein was −5.54. The model reliability was further checked by ERRAT [45] that analyzes the statistics of nonbonded interactions between different atom types and plots the value of the error function versus position of a 9-residue sliding window as calculated by a comparison with statistics from highly refined structures. The ERRAT results showed 84.648 overall model quality (Figure 8). The *Z*-scores, Ramachandran plot, and ERRAT results confirmed that the quality of the RABVG trimer model is suitable for future theoretical studies.

**Figure 9:**
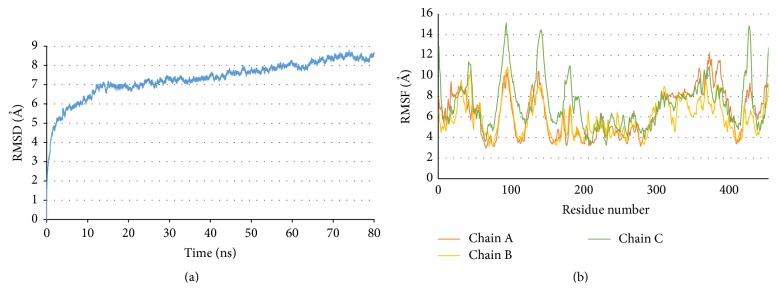
(a) RMSD of RABVG trimer at 310 K. (b) The RMSF (root mean square fluctuations) per residue per chain of RABVG trimer from 12000 to 8000 frames.

Molecular dynamics (MD) simulations were carried on RABVG trimer at 310 K. From these theoretical studies we retrieved the RMSD values from C*α* atoms. This suggests that the system reached structural stability and simulation integrity. The magnitude of the RMSD (7 Å) indicates that the RABVG trimer is stabilized at 12 ns and remains constant until the end of the simulation (Figure 9).

On the other hand, the residues we used in the root mean square fluctuations (RMSF) identify the regions responsible for the fluctuations during the MD simulations. The areas with higher fluctuations correspond to beta-loop-beta chains residues and turn regions. Those with lower fluctuations are regions of *α*-helices. During the MD simulation, no structural uncoiling was observed. At 310 K, the RMSF values go from 3 to 15 Å obtained from 12000 to 7500 frames; RMSF are similar for all chains except Ala87 to Thr100, Pro136 to Thr147, Phe173 to Asn201, and Ser422 to Val435 residues of chain C (Figure 9).

The regions with the most fluctuations have not been described as important areas to maintain the structural stability of the G protein trimer. This does not generate significant changes within the main trimer binding regions.

**Figure 10:**
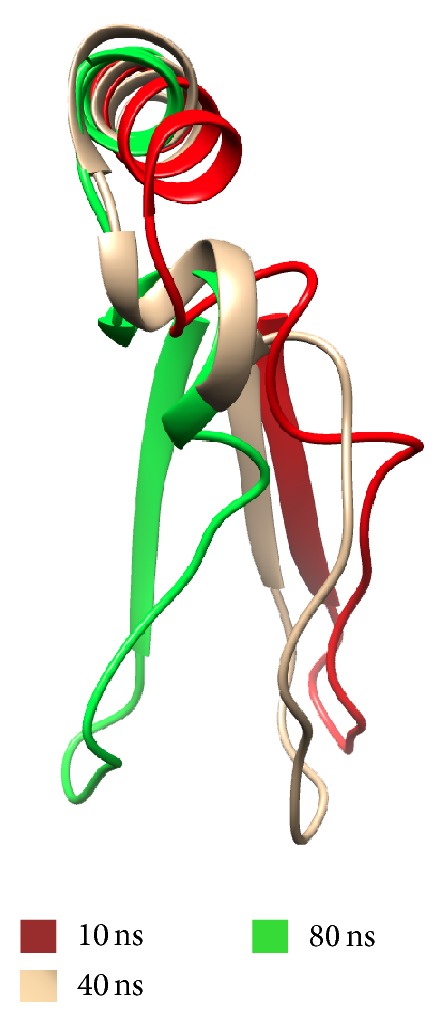
Chain C conformational chance during molecular dynamics. Residues 125 to 131 (PRYSEEL). Red 10 ns, beige 40 ns, and green 80 ns.

In the residues 125 to 131 conformational change of chain C after 10 ns was detected and is stable from 40 ns to 80 ns when molecular dynamic end (Figure 10).

During molecular dynamic simulations, the structure of the G protein remained stable after 12 ns. It maintained the trimer bound and conserved key interactions to maintain the stability of the structure.

## 4. Conclusions

The molecular modeling of G protein was performed by a I-TASSER server and was evaluated via a Rachamandran plot and ERRAT program obtained 84.64% and 89.9% of the residues in the favorable regions and overall quality factor, respectively.

The interactions between residues, 274 to 293, are directly linked to the structure of the prefusion and postfusion of Glycoprotein. These interactions are important to maintain these structures.

This is important for structural stability of the G protein trimer. It might be a good target for antiviral compounds because such modifications would change the helical conformation and be detrimental to the virus.

The fluctuations that occurred during the molecular dynamics do not affect the stability of the structure of G protein trimer. Protein G structural stability was obtained by molecular dynamics analysis at 12 ns.

## Figures and Tables

**Table 1:** Predicted disulfide bonds.

Region	Predicted bonds
17–479	LVFSLCFGKFP–IFLMTCCRRVN
43–271	IHHLSCPNNLV–DETKWCPPDQL
54–226	VEDEGCTNLSG–KGSKTCGFVDE
80–188	VNGFTCTGVVT–VSSTYCSTNHD
113–480	PTPDACRAAYN–FLMTCCRRVNR
178–208	FPSGKCSGITV–RLGTSCDIFTN
242–370	SLKGACKLKLC–RVGGRCHPHVN
247–363	CKLKLCGVLGL–IPSKGCLRVGG
